# A Machine Learning Approach to Gene Expression in Hypertrophic Cardiomyopathy

**DOI:** 10.3390/ph17101364

**Published:** 2024-10-12

**Authors:** Jelena Pavić, Marko Živanović, Irena Tanasković, Ognjen Pavić, Vesna Stanković, Katarina Virijević, Tamara Mladenović, Jelena Košarić, Bogdan Milićević, Safi Ur Rehman Qamar, Lazar Velicki, Ivana Novaković, Andrej Preveden, Dejana Popović, Milorad Tesić, Stefan Seman, Nenad Filipović

**Affiliations:** 1 Institute for Information Technologies Kragujevac, University of Kragujevac, 34000 Kragujevac, Serbia; zivanovicmkg@gmail.com (M.Ž.); opavic@kg.ac.rs (O.P.); msc.katarina.virijevic@gmail.com (K.V.); tamara.mladenovic@uni.kg.ac.rs (T.M.); jelena.kosaric@hotmail.com (J.K.); bogdan.milicevic@uni.kg.ac.rs (B.M.); 2 Faculty of Engineering, University of Kragujevac, 34000 Kragujevac, Serbiafica@kg.ac.rs (N.F.); 3 Department of Histology and Embryology, Faculty of Medical Sciences, University of Kragujevac, 34000 Kragujevac, Serbia; irena.vuk@gmail.com; 4 Department of Pathology, Faculty of Medical Sciences, University of Kragujevac, 34000 Kragujevac, Serbia; wesna.stankovic@gmail.com; 5 Bioengineering Research and Development Center (BioIRC), 34000 Kragujevac, Serbia; 6 Faculty of Medicine, University of Novi Sad, 21000 Novi Sad, Serbia; lazar.velicki@mf.uns.ac.rs (L.V.); andrej.preveden@mf.uns.ac.rs (A.P.); 7 Institute of Cardiovascular Diseases Vojvodina, 21204 Sremska Kamenica, Serbia; 8 Faculty of Medicine, University of Belgrade, 11000 Belgrade, Serbia; ivana.novakovic@med.bg.ac.rs (I.N.); misa.tesic@gmail.com (M.T.); 9 Department of Cardiovascular Diseases, Mayo Clinic, Rochester, MN 55905, USA; dejana.popovic99@gmail.com; 10 Clinic for Cardiology, University Clinical Center of Serbia, 11000 Belgrade, Serbia; 11 Faculty of Sports and Physical Education, University of Belgrade, 11000 Belgrade, Serbia; sseman@outlook.com

**Keywords:** hypertrophic cardiomyopathy, apoptosis, *CASP3*, *BCL2*, gene expression

## Abstract

Background/Objectives: Hypertrophic cardiomyopathy (HCM) is a common heart disorder characterized by the thickening of the heart muscle, particularly in the left ventricle, which increases the risk of cardiac complications. This study aims to analyze the expression of apoptosis-regulating genes (*CASP8*, *CASP9*, *CASP3*, *BAX*, and *BCL2*) in blood samples from HCM patients, to better understand their potential as biomarkers for disease progression. Methods: Quantitative real-time PCR (qPCR) was used to evaluate gene expression in blood samples from 93 HCM patients. The correlation between apoptosis-regulating genes was conducted and clinical parameters were integrated for feature importance and clustering analysis. Results: Most patients exhibited significant downregulation of *CASP8*, *CASP9*, and *CASP3*. In contrast, BAX expression was elevated in 71 out of 93 patients, while *BCL2* was increased in 55 out of 93 patients. Correlation analysis revealed weak negative correlations between the *BAX*/*BCL2* ratio and *CASP* gene expression. Conclusions: These findings suggest that reduced expression of apoptotic genes may indicate a protective cellular mechanism, which could serve as a biomarker for disease progression. Further studies are needed to investigate the potential for therapeutic modulation of these pathways to improve patient outcomes.

## 1. Introduction

Cardiovascular diseases (CVDs) are the leading cause of death, with significant variations in prevalence across different populations and geographic areas [1,2]. As a major healthcare burden worldwide [3], CVDs encompass a group of conditions characterized by heart and blood vessel damage [4], including coronary artery disease, hypertension, heart failure, heart valve disorders, and others [5]. Among these conditions is cardiomyopathy, a subset of heart diseases described as disorders of the heart muscle [6], whereby the muscle becomes weak and inelastic and can either widen or thicken, depending on the specific type of disease. As a result, the heart’s ability to maintain efficient blood flow is reduced [7,8]. Following the European Society of Cardiology (ESC), cardiomyopathies are classified as either dilated, hypertrophic, restrictive, or unclassified, each with distinct characteristics and implications for cardiac function and patient health [9,10]. In the present study, our focus is on hypertrophic cardiomyopathy (HCM).

HCM is defined by an abnormal thickening of the heart muscle (myocardium), which particularly affects the left ventricle [11]. Due to this thickening, the heart pumps blood more slowly, which can cause a complete blockage of blood flow to the heart, leading to death [12]. Hypertrophy most often affects the wall between the left and right ventricles (interventricular septum) [13]. The cause of its occurrence is still unknown, but it is considered congenital because it runs in the family and is inherited autosomal dominant [9,14]. Symptoms include shortness of breath (dyspnea), chest pain, fainting, and an increased risk of cardiac arrhythmias [15,16]. It is the most common form of cardiomyopathy and its variability in clinical manifestations, etiology, and disease progression makes it a suitable target for various research studies [17,18]. Cardiomyopathies occur in different age groups, including children and adolescents [19]. In this regard, it is of great importance to understand the molecular mechanisms that are essential to the onset and progression of this disease [20]. Such knowledge can provide us with an insight into the pathophysiology of the disease [21].

Programmed cell death, known as apoptosis, is a specific form of cell death that occurs as a normal physiological process within the cell and is crucial for the maintenance of homeostasis [22,23]. When DNA damage occurs, cell repair mechanisms either correct the damage or initiate apoptosis to prevent the propagation of genetic mutations [24]. Dysregulation of these pathways can lead to various pathological conditions, including cancer. Moreover, the immune system can trigger apoptosis in response to the presence of some viruses, pathogens, and various stressors such as oxidative stress and radiation [25]. During apoptosis, cells undergo morphological and biochemical changes, including cell shrinkage, membrane blebbing, chromatin condensation, and DNA fragmentation [23]. Apoptosis can be initiated through the intrinsic (mitochondrial) pathway and extrinsic (death receptor) pathway [26]. The extrinsic pathway is activated by the binding of ligands to death receptors from the TNF/Fas family on the cell surface. The binding of the Fas ligand to the Fas receptor (CD95) leads to the formation of a complex called the DISC, which consists of various proteins. Within this DISC complex, caspase-8 becomes an active enzyme by switching from the inactive form of procaspase-8 [27]. Activated casp-8 can directly activate caspase-3, leading to the degradation of cellular components and ultimately cell death. This external pathway plays a key role in the elimination of harmful cells [26,28]. The intrinsic pathway, also known as the mitochondrial, is triggered by internal signals such as DNA damage or oxidative stress, resulting in the release of cytochrome c from the mitochondria into the cytoplasm, forming an apoptotic complex with APAF-1 and the proenzyme of caspase-9 (procaspase-9). When cytochrome c binds to this protein, it undergoes conformational changes and creates apoptosomes that activate caspase-9. Caspase-9 also triggers a cascade of reactions that lead to the activation of effector Caspase-3, which causes the breakdown of cell proteins and DNA [29,30].

Caspases, a family of cysteine proteases, are proteins that function as enzymes that play a key role in regulating and executing apoptosis [31,32]. They are located in the cytoplasm as inactive precursor molecules (proenzymes) and are activated under certain conditions, such as DNA damage or external stress, leading to the breakdown of proteins and cell death. Caspases are divided into two groups: one is responsible for the initiation of apoptosis (caspases-2, 8, 9, and 10), while the other includes effector caspases (caspases-3, 6, and 7) that are involved in the final phase of apoptosis [33,34]. In the previously discussed processes of cell death, a whole range of regulatory proteins can modulate the manifestation of this process, either through activation/suppression. Bcl-2-associated X-protein (BAX) is a pro-apoptotic protein that belongs to the Bcl-2 family of proteins. It promotes apoptosis by facilitating the permeability of the mitochondrial membrane, which leads to the release of cytochrome c and the activation of the caspase cascade that initiates cell death. B-cell lymphoma 2 (Bcl-2) is an anti-apoptotic protein that inhibits apoptosis by binding and inactivating pro-apoptotic proteins such as BAX, preventing mitochondrial membrane permeabilization and temporally inhibiting the process of apoptosis [35]. It is thought that the balance between their activity levels, rather than the amount of each protein individually, plays a crucial role in determining how likely the cell is to undergo apoptosis [36]. In this paper, we will analyze the expression of *CASPs* 3, 8, and 9, and the *BAX/BCL2* ratio, which are all crucial for the cell death process.

Numerous studies have investigated the molecular mechanisms of HCM, mostly focusing on structural proteins or metabolic dysfunctions in cardiomyocytes [37,38], but the exact molecular mechanisms that eventually lead to the clinical presentation of HCM remain unclear [39,40]. Fewer studies investigated role of apoptosis regulators, focusing on the expression of *CASP* genes and the balance between pro-apoptotic and anti-apoptotic factors such as the *BAX*/*BCL2* ratio, in the pathophysiology of HCM [41,42].

This study analyzes the expression of key apoptosis-regulating genes, specifically *CASPs* 3, 8, and 9, *BAX*, and *BCL2*, in blood samples of patients with HCM. By investigating the association between the expression levels of these genes and the clinical manifestations of HCM, the study claims to identify potential biomarkers for disease progression. In addition, this study aims to apply various methods, including feature importance and correlation analysis, to create visual representations that will help us better understand the underlying mechanisms. This approach is intended to provide clearer insights into gene expression profiles, thus facilitating the classification of patients, the personalization of therapeutic strategies, and improving diagnostic accuracy.

## 2. Results

A total of 93 patients were included in the study. The baseline clinical characteristics of the study population are presented in Supplementary Table S1. Genetic testing was conducted on blood samples collected during the initial visit, and the results presented in the corresponding figures were obtained using these samples. The second visit served as a follow-up to monitor any changes in patients’ clinical characteristics over time. The mean interventricular septum thickness was 18.5 ± 3.5 mm at Visit 1 and 18.9 ± 3.6 mm at Visit 2, while posterior wall thickness averaged 12.7 ± 3.8 mm and 12.4 ± 3 mm, respectively. Left atrium diameter remained stable between visits, with a mean of 42.2 ± 5.1 mm at Visit 1 and 42.6 ± 5.1 mm at Visit 2. Left ventricular ejection fraction (EF) was preserved in the majority of patients, with a mean of 65 ± 7.6% at Visit 1 and 64.8 ± 6.8% at Visit 2.

Notably, N-terminal pro B-type Natriuretic Peptide (NT-proBNP) levels were lower at Visit 2, with a mean of 1702 ± 1329 pg/mL compared to 2240 ± 1398 pg/mL at Visit 1. Troponin concentrations also decreased slightly from 33.5 ± 90.8 ng/L at Visit 1 to 30 ± 66.8 ng/L at Visit 2. The incidence of atrial fibrillation increased slightly between visits, from 10 patients at Visit 1 to 12 patients at Visit 2. Syncope was present in 8 patients at Visit 1 and 2 patients at Visit 2.

New York Heart Association (NYHA) classification showed a similar distribution across visits, with most patients classified as NYHA class I or II. The left ventricular outflow tract maximum pressure gradient (LVOT maxPG) showed a slight increase from 15.6 ± 23.3 mmHg to 16.6 ± 22.6 mmHg between visits.

### 2.1. Gene Expression Analysis

**Figure 1:**
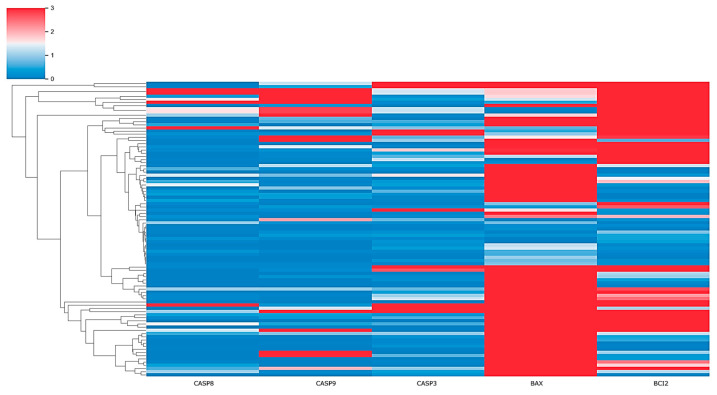
Heatmap showing expression of selected genes across patient samples. The color intensity represents the level of gene expression: bright red indicates high expression; white indicates average expression; light-blue indicates low expression. Rows correspond to individual patients and columns represent specific genes (*CASP8*, *CASP9*, *CASP3*, *BAX*, and *BCL2*).

The expression analysis of all previously described samples for *CASP8*, *CASP9*, *CASP3*, *BAX*, and *BCL2* genes is shown in the following heatmap (Figure 1).

Based on the heatmap and qPCR results, it appears that the majority of the *CASP8* gene samples are predominantly colored blue, indicating lower expression levels compared to reference conditions. Specifically, 79/93 patients showed reduced *CASP8* expression. Similarly, 72/93 patients showed decreased *CASP9* expression and 74/93 patients demonstrated lower *CASP3* expression, highlighting a consistent downregulation of these apoptotic genes across the patient cohort.

*BAX* showed increased expression in the majority of patients (71/93), while *BCL2* showed increased expression in almost half of the patients (55/93), as shown in the graphic. These results will be further analyzed in the Section 3. In 10/93 patients, the *BAX*/*BCL2* ratio was relatively low, which may indicate a dominant anti-apoptotic activity (i.e., more *BCL2* than *BAX*), while in 27/93 patients, *BAX*/*BCL2* ratio is high (i.e., more *BAX* than *BCL2*).

### 2.2. Correlation between the BAX/BCL2 Ratio and CASP Genes

**Figure 2:**
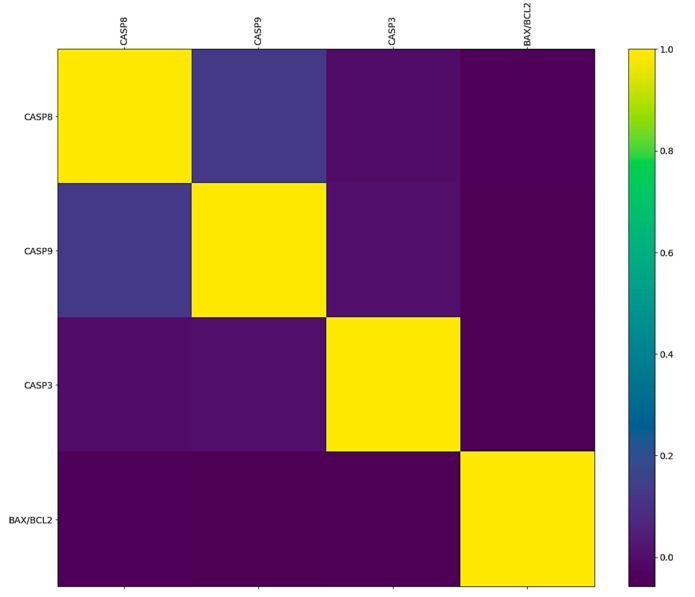
Correlation between the *BAX*/*BCL2* ratio and *CASP* genes.

The correlation between the *BAX*/*BCL2* ratio and expression of *CASP8*, *CASP9*, and *CASP3* genes is shown in Figure 2.

Correlation analysis between the *BAX*/*BCL2* ratio and *CASP8*, *CASP9*, and *CASP3* gene expression showed very weak and negative correlations. More precisely, the correlation between the *BAX*/*BCL2* ratio and *CASP8* is −0.041, while the correlation with *CASP9* is −0.057, and with *CASP3* is −0.049. There is no strong linear relationship between these genes in a sample of patients with HCM.

### 2.3. The Feature Importance of Analyzed Apoptotic Genes

Feature importance analysis was performed using methods from the scikit-learn library applied to Random Forest and Extreme Gradient Boosted Trees models, enabling the identification of the most influential genes contributing to the observed differences. Genes typically demonstrate significant importance when analyzed alongside clinical data. The highest importance compared to other genes exhibited *BCL2* (12.0). Importance analyses of the genes *CASP8* and *CASP3* showed equal results (7.0), while *CASP9* displayed slightly lower importance (6.0). The lowest feature value was observed for the *BAX* gene (5.0). This distribution of gene importance suggests their relative prevalence in apoptosis in the blood samples of HCM patients. This could be interesting for the further examination of potential predictive biomarkers.

### 2.4. Risk Profile in HCM Patients through Clustering Analysis

**Figure 3:**
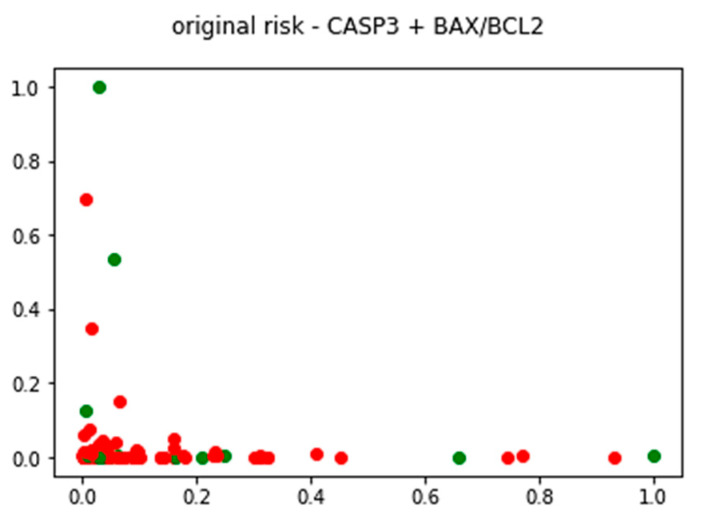
Ground truth state of HCM patients expressed by apoptotic genes (the red points represent high-risk patients, while the green points represent low-risk patients).

**Figure 4:**
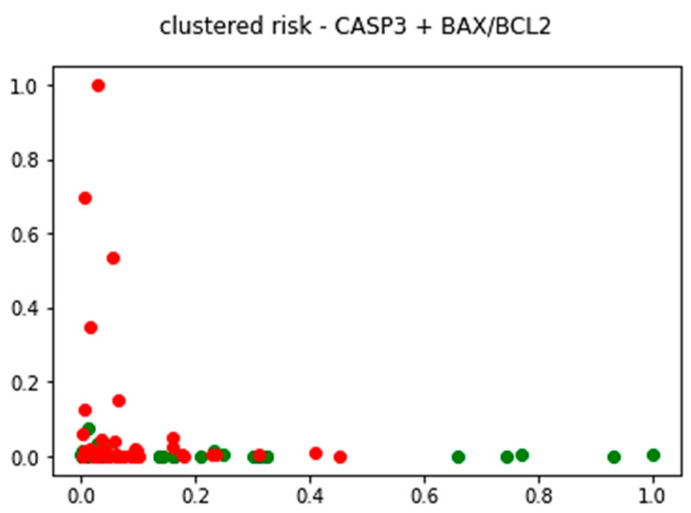
K-means clustered HCM patients expressed by apoptotic genes (the red points represent high-risk patients, while the green points represent low-risk patients).

Further, an unsupervised analysis was performed using a k-means clustering model created to examine the effect of gene expression on outcomes, without conditioning the results on any prior external knowledge (Figure 3 and Figure 4).

The comparison between the ground truth risk classes and the results of unsupervised clustering, using apoptotic genes, shows a fair degree of similarity when comparing the expected and the achieved high-risk classes. In the context of the available dataset, the k-means clustering model was able to correctly identify 63.1% of high-risk patients as belonging to a single group using only apoptotic genes for model training. When the same analysis was conducted using other genes involved in oxidative stress and pathogenesis of the disease, the final clusters were too heterogeneous with regards to risk class association to be useful for risk prediction. Even though the overall prediction accuracy of such clustering models based solely on genetic data was much lower than classification models trained using clinical data, their performance using a very low number of predictors serves as a good basis for future research of early sign recognition and early diagnosis of HCM.

## 3. Discussion

Apoptosis in cardiomyocytes is triggered by various stress factors commonly associated with CVD, such as increased cytokine production, oxidative stress, and DNA damage. This tightly regulated cell death process can be protective for the heart because inhibition of apoptosis has been shown to have cardioprotective effects under certain conditions. The crucial significance of apoptosis inhibition and blocking the initiation of cell death in cardiomyocytes is of vital importance because cardiomyocytes are irreversibly postmitotic terminally differentiated cells [43,44].

Stressed cardiomyocytes secrete inflammatory cytokines, chemokines, cell adhesion proteins, and danger-associated molecular patterns (DAMPs) [43], as well as modulate the expression of pro-apoptotic and anti-apoptotic proteins like *BAX* and *BCL2* [45]. These molecules stimulate immune responses, monocytes, and lymphocytes, further amplifying the inflammatory microenvironment in the heart. These substances enter the bloodstream through various mechanisms, released from stressed or damaged cardiomyocytes. The appearance of these molecules in the bloodstream could be significant for monitoring disease progression and could potentially serve as diagnostic biomarkers in heart conditions such as HCM.

Our study included samples from 93 patients with HCM, in which whole blood samples were analyzed for the expression of *CASP8*, *CASP9*, and *CASP3* genes, as well as their relationship to *BAX*/*BCL2*. Using qPCR, we measured gene expression levels and analyze the potential correlation between these genes and the clinical progression of HCM. The heatmap and qPCR results revealed a consistent downregulation of the apoptotic initiators *CASP8* and *CASP9*, and the executioner *CASP3* in the majority of patients. Specifically, 79/93 patients showed reduced *CASP8* expression. Similarly, 72/93 patients showed decreased *CASP9* expression and 74/93 patients demonstrated lower *CASP3* expression, highlighting a consistent downregulation of these apoptotic genes across the patient cohort.

The lower *CASP8*, *CASP9*, and *CASP3* expression in most patients in our study indicates that these genes have reduced activity, which may indicate inhibition of apoptosis in the given samples. These results may suggest that both intrinsic and extrinsic activation pathways are inactive, possibly due to an activation mechanism to avoid excessive cell death. If this downregulation of *CASP8*, *CASP9*, and *CASP3* genes has a protective role, modulation of this pathway should be carefully considered as a potential therapeutic option.

Furthermore, the pro-apoptotic gene *BAX* showed increased expression in the majority of patients (71/93), while *BCL2*, an anti-apoptotic gene, showed increased expression in almost half of the patients (55/93). This could indicate the activation of mechanisms to remove damaged or dysfunctional cells, since *BAX* is a pro-apoptotic gene and promotes apoptosis.

Correlation analysis between the *BAX*/*BCL2* ratio and *CASP8*, *CASP9*, and *CASP3* gene expression showed very weak and negative correlations, suggesting that there is no strong linear or proportional association between these factors in our patient sample. More precisely, the correlation between the *BAX*/*BCL2* ratio and *CASP8* is −0.041, while the correlation with *CASP9* is −0.057, and with *CASP3* is −0.049. All these correlations are negative but statistically negligible. These findings indicate that the apoptotic markers are largely independent of one another in the context of HCM, underscoring the complexity of apoptotic regulation in this disease.

By applying K-means clustering, we classified our patients based on their apoptotic gene expression profiles. We demonstrated a 63.1% success rate in identifying high-risk patients based on apoptotic gene expression. This result highlights the potential of apoptotic markers as predictive tools for risk stratification in HCM. Importantly, clustering models incorporating additional genes related to oxidative stress and inflammation did not achieve the same degree of accuracy, suggesting that apoptotic gene expression alone carries significant predictive weight.

Feature importance analysis performed using the scikit-learn library applied to Random Forest and Extreme Gradient Boosted Trees models revealed the most influential genes contributing to the observed differences. The highest importance compared to other genes exhibited *BCL2*.

Previous research has shown that *BCL2* is a key anti-apoptotic protein whose increased expression can increase cell resistance to apoptosis, with the *BCL2*/*BAX* ratio acting as a regulator that balances cell death. These studies have shown that inhibition of apoptosis through increasing the *BCL2*/*BAX* ratio contributes to the survival of cardiomyocytes in the peri-infarct area after myocardial infarction and reperfusion (MI/RI), reducing the degree of cardiac damage and preventing the opening of the mitochondrial permeability pore due to hypoxia [46].

On the other hand, Latif et al. demonstrated a notable increase in the pro-apoptotic protein *BAX* in patients with heart failure, which is in agreement with the results of our studies. According to the same data, elevated *BAX* levels facilitate the release of cytochrome c from mitochondria and accelerate the opening of voltage-dependent anion channels, thereby promoting apoptosis in cardiomyocytes [47]. Oxidative stress enhances the transcriptional activity and accumulation of the P53 protein, which induces apoptosis in cardiomyocytes by stimulating *BAX* expression or inhibiting *BCL2* expression [48].

All the examined signaling pathways in our study, especially the genes that regulate them, are interconnected and have a common effect on the potential death of cardiomyocytes. Numerous studies aimed at discovering new drugs and improving therapeutic procedures are based on the modulation of these signaling pathways to achieve apoptosis regulation and promote cardiomyocyte survival [49]. Available literature and data show, for example, that melatonin activates the JAK2-STAT3 prosurvival signaling pathway. This reduces cardiomyocyte apoptosis and protects against myocardial ischemia by upregulating *BCL2* expression while downregulating *BAX* and *CASP3* [50] or, for example, trimetazidine (TMZ), which was found to reduce the *BAX*/*BCL2* ratio and *CASP3* expression and inhibit cardiomyocyte apoptosis induced by reperfusion injury (I/R) by activating the Akt signaling pathway [51].

The results of our study showed that *BAX* was elevated in most patients, while *BCL2* was elevated in half of the analyzed patients, which suggests that in 55 of the 93 patients analyzed in our study, elevated *BCL2* prevented the release of cytochromes with the subsequent activation of *CASP3*, which led to the inhibition of apoptosis. This may indicate that the cells are “trying” to survive despite the sarcomeric gene mutation in cardiomyocytes shown in numerous studies [52,53,54,55].

Apoptosis of cardiomyocytes with triple protein gene mutations in sarcomeres would be life-threatening, but, on the other hand, their survival via the inhibition of apoptosis leads to left ventricular hypertrophy, increased myocardial contractility, diastolic dysfunction, myofibrillar disarray, and fibrosis [56,57,58].

## 4. Materials and Methods

### 4.1. Materials 

The experiment was performed with different reagents obtained from different manufacturers. For RNA isolation, Trizol (TRI Reagent) was purchased from Sigma-Aldrich, Steinheim, Germany. Phosphate-buffered saline (PBS) was obtained from Capricorn Scientific, Germany. PCR-grade isopropanol and ethanol were obtained from Thermo Fisher, Waltham, MA, USA. Chloroform was from Alfa Aesar, USA (Thermo Fisher Scientific). Primers for the PCR reaction was purchased from Thermo Fisher, Waltham, MA, USA. The reverse transcription kit was the EurX NG dART RT kit (eurX Genetics EUROPE, Gdańsk, Poland). A PCR kit from Promega GoTak™ kPCR Master Mix (Promega, Madison, WI, USA) was used to determine relative gene expression. The genomic DNA isolation kit was from the PurelinkTM Genomic DNA Kit (Thermo Fisher Scientific, Waltham, MA, USA). The kit for polymorphism determination was from TakMan SNPs Genotyping Assay (Applied Biosystems, Waltham, MA, USA).

### 4.2. Blood Sampling

Whole blood samples were collected from 93 patients (57 men (m) and 36 women (f)) diagnosed with HCM (Table 1). The controls were slightly younger and without any medical record. A 2 mL whole blood sample was collected over time using a blood Vacutainer. In our study, the first visit represents the original consultation where blood samples were collected. This initial sampling allowed us to analyze the expression of key genes based on the primary clinical evaluation. The second visit served completely as a follow-up, intended for medical practitioners to monitor any changes in the patient’s condition for therapeutic purposes. Blood samples were also collected from healthy individuals, serving as controls, alongside the two patient groups from the two clinical centers. Medical records containing information such as age, sex, NYHA classification, symptoms, family history, and more information on patients’ conditions were collected from the hospital database. Samples were collected from the University Clinical Centre of Serbia and the Institute of Cardiovascular Diseases, Sremska Kamenica. This study was approved by the local ethics committee of the Institute of Cardiovascular Diseases, Sremska Kamenica (Decision No.: 1813-1/4), and the University Clinical Centre of Serbia, Clinic for Cardiology (Decision No.: 2551-01/11). An aliquot of whole blood was stored at −20 °C. After pooling all samples, total RNA was isolated.

### 4.3. Analysis of Gene Expression by Quantitative Real-Time Polymerase Chain Reaction (qRT-PCR)

RNA isolation is a crucial step in preparing samples for gene expression analysis by qRT-PCR.

#### 4.3.1. Isolation of RNA

Total RNA was isolated manually using Trizol reagent according to the instructions of Chomczynski and Sacchi (1987) [59]. Aliquots of frozen blood samples were left in a water bath for 20 min to thaw. After the samples were thawed, 1 mL of blood and 2 mL of PBS were added to the tube and everything was centrifuged (MPW-150R, Warsaw, Poland) at 10,000× *g* (RCF) for 5 min. The supernatant was discarded, 500 µL of Trizol was added, and the pellet was well resuspended. After incubation at room temperature, the samples were centrifuged at 1000× *g* (RCF) for 5 min. After, 100 µL of chloroform was added to the supernatant and the microtubes were well vortexed for 15 s. After holding at room temperature for 2–3 min, the samples were centrifuged at 15,000× *g* (RCF) for 15 min at 4 °C. Centrifugation separates 3 phases: RNA is found in the higher clear phase. The upper aqueous phase was transferred to a new microtube and the RNA was precipitated using 250 µL of isopropanol followed by incubation for 10 min at room temperature. Precipitated RNA was centrifuged at 12,000× *g* (RCF) for 10 min at 4 °C. The supernatant was discarded and the pellet was washed with 80% ethanol twice by centrifugation at 7500× *g* (RCF) for 5 min at 4 °C. The supernatant was removed and the RNA pellet was allowed to dry in a dry bath (DLAB HB120-S Dry Bath, Beijing, China and then resuspended in 30 μL of RNase-free water. Total RNA was measured on Thermo Scientific™ μDrop™ and μDrop Duo Plates (Thermo Fisher, Waltham, USA) and kept at −80 °C until the next step.

#### 4.3.2. Reverse Transcription (RT-PCR)

Total RNA was converted into complementary DNA (Complementary DNA—cDNA) using the NG dart Reverse Transcription Kit in a volume of 20 µL per reaction according to the manufacturer’s instructions. The reaction mixture contained NG dART RT Mix, 5× NG cDNA buffer, Oligo(dT) and Randomm hexamers using the following thermal cycling temperatures: 30–60 min at 50 °C, 25 °C for 10 min, and then 85 °C for 5 min. After this reaction, the concentration was measured for each sample and aliquots of cDNA were stored at −80 °C until a quantitative polymerase chain reaction was performed.

#### 4.3.3. Quantification of Relative Gene Expression (qPCR)

We performed qPCR analysis using the Mic qPCR Cycler according to the MIQE guidelines. A volume of 20 µL in a reaction mixture containing GoTaq^®^ qPCR Master Mix (2×), Forward Primer, Reverse Primer (200 nM–1 μM), and nuclease-free water was used to perform qPCR. The reaction mixture contained the components shown in Supplementary Table S2. NTC (No Template Control) with all components except the sample was used as a negative control. Amplification conditions were as follows: an initial step of double-stranded DNA denaturation at a temperature of 95 °C for 2 min, followed by binding of specific oligonucleotides—primers (annealing), and primer extension at a temperature of 60 °C for 60 s (English extension). These three steps were repeated up to 40 times, resulting in a large number of DNA copies. After 40 cycles, the melting curve of the PCR reaction product was analyzed.

The following apoptosis primers were used for the reaction: *CASP8*, *CASP9*, *CASP3*, *BAX*, and *BCL2*.

The obtained results were analyzed with the Mic qPCR software and cycle threshold values (Δ*CT*) were read and calculated using the formula 2^−ΔΔCT^ used to express the differences between the observed genes and the gene expression of Beta-actin (*ACTB*, “housekeeping” gene) described in the available literature [60,61]. Results are presented as the mean of duplicates of each sample, and relative mRNA expression levels for each sample are presented as the ratio of expression of the gene of interest to the housekeeping gene, which was used for normalization.

The following formula was used to calculate relative gene expression from blood samples:

$$2^{-\Delta \Delta CT}=\Delta CT_{1}-\Delta CT_{2}$$

where $\Delta CT_{1}$ = CT value of the gene of interest in the sample, $\Delta CT_{2}$ = CT value of *ACTB* in the sample.

#### 4.3.4. Statistical Analysis

Gene expression analysis was performed in duplicate and data are expressed as mean ± standard error. To obtain statistical significance, ANOVA was used for multiple comparisons and Dunnett’s test was used to compare individual groups with control. Gene expression results were processed in the SPSS (Chicago, IL, USA) statistical analysis program (SPSS for Windows, ver. 17, 2008). In addition, correlation analysis was conducted to examine the relationships between gene expression levels using Python 3.8 libraries Pandas (version 2.2) and NumPy (version 1.21) for linear and non-linear correlation calculation, respectively. Feature importance analysis was performed using methods from the scikit-learn library applied to Random Forest and Extreme Gradient Boosted Trees models, enabling the identification of the most influential genes contributing to the observed differences. In addition, a k-means clustering model was created in order to conduct unsupervised analysis, allowing for the grouping of samples based on gene expression profiles. Prior to clustering, outlier removal and data scaling were applied to ensure that all genes were on the same scale and that those scales were not swayed by a minority of extreme cases. Additionally, Principal Component Analysis (PCA) was performed for dimensionality reduction, to ensure that the results could be presented in a two-dimensional space and plotted in a format conductive to human analysis.

The Python script generates a clustered heatmap using hierarchical clustering and visualization libraries such as matplotlib and seaborn. Gene expression data are loaded from a CSV file into a panda’s data frame, where rows correspond to genes and columns to samples. The resulting heatmap visualizes the hierarchical structure of the genes alongside the expression values, which are represented by color intensity within a specified range (vmin = 0, vmax = 3).

## 5. Conclusions

In this study, we analyzed the expression of key apoptosis-regulating genes—*CASP8*, *CASP9*, *CASP3*, *BAX*, and *BCL2*—in the blood samples of patients with HCM. We aimed to investigate the association between gene expression levels and the clinical manifestations of HCM, applying methods such as feature importance and correlation analysis to uncover potential biomarkers for disease progression. Through these analyses, we tried to enhance understanding of the molecular mechanisms involved in HCM, by providing clearer insights that could facilitate patient classification, personalize therapeutic strategies, and improve diagnostic accuracy. Our findings suggest that patients with significantly reduced expression of *CASP3* and *CASP8* may exhibit a less active apoptotic response, potentially correlating with worse prognosis. These patients might benefit from therapies aimed at increasing apoptotic activity in heart tissue or blood, particularly to target the removal of damaged cells. Conversely, the increased expression of the anti-apoptotic gene *BCL2* in some patients indicates that inhibition of this gene could be a viable therapeutic strategy to prevent the accumulation of damaged cells, which may contribute to the decline of cardiac function.

Overall, changes in the expression of *CASP8*, *CASP9*, *CASP3*, *BAX*, and *BCL2* in the blood could serve as valuable biomarkers for identifying and monitoring patients with HCM. If further validated in larger cohorts, these biomarkers could potentially aid in the early detection and ongoing monitoring of HCM, providing a foundation for the development of targeted therapies and improving patient outcomes.

## Figures and Tables

**Table 1:** Patient characteristics.

Location	Patients	Sex (F/M)	Age	NYHA Class (I/II/III)
Group 1	43	13/30	60.4 ± 10.3	19/20/4
Group 2	50	22/28	53.5 ± 13.1	30/18/2

## Data Availability

The authors declare that all data supporting the findings are available within the paper or from the authors upon request.

## Supplementary Materials

The following supporting information can be downloaded at: https://www.mdpi.com/article/10.3390/ph17101364/s1. Table S1. Overview of Baseline Clinical Parameters; Table S2. Primers used in qRT-PCR analysis.
