# Supplementary material for: A Machine Learning Approach to Gene Expression in Hypertrophic Cardiomyopathy
Source: Pharmaceuticals (Basel). 2024 Oct 12;17(10):1364. doi: 10.3390/ph17101364 (PMC11510441; doi:10.3390/ph17101364)
Supplement: Supplementary file 1 [file pharmaceuticals-17-01364-s001.zip › pharmaceuticals-3252628-supplementary.pdf]

Supplementary Table S1. Overview of Baseline Clinical Parameters

**Variable**	**Visit 1**	**Visit 2**
Syncope (present/absent)	8/85	2/91
NYHA class (I/II/III)	49/38/6	50/40/3
Interventricular septum thickness	18.5±3.5	18.9±3.6
Posterior wall thickness	12.7±3.8	12.4±3
Left atrium diameter	42.2±5.1	42.6±5.1
Ejection Fraction	65±7.6	64.8±6.8
LVOT maxPG	15.6±23.3	16.6±22.6
NT-BNP	2240±1398	1702±1329
Troponin	33.5±90.8	30±66.8
Atrial fibrillation (present/absent)	10/83	12/81
LVIDs	29.1±5.9	28.7±5.7
LVIDd	46.9±5.5	47.3±5.3

***NYHA**, New York Heart Association; **LVOT maxPG**, Left ventricular outflow tract pressure gradient maxPG; **NT-BNP**, N-terminal pro B-type Natriuretic Peptide; **LVIDs**, Left ventricular internal diameter end systole; **LVIDd**, Left ventricular internal diameter end diastole.*

Supplementary Table S2. Primers used in qRT-PCR analysis

**Primers**	**Abbreviation**	**Sequences**
Caspase 8	CASP8	F 5′ GATAACGGAGGCTGGGATGC 3′
		R 5′ GACTTCACTTGTGGCCCAGAT 3′
Caspase 9	CASP9	F 5′ TGGAAATAAACTGCACCCGGA 3′
		R 5′ TCCTTTCTCTTCACCCAAACA 3′
Caspase 3	CASP3	F 5′ GCTTCAGGGTTTCATCCAGGA 3’
		R 5′ CAATCATCCTCTGCAGCTCCA 3′
Bcl-2 Associated X-protein	BAX	F 5′ GCTTCAGGGTTTCATCCAGGA 3′
		R 5′ CAATCATCCTCTGCAGCTCCA 3′
B-cell lymphoma 2	BCL2	F 5′ GATAACGGAGGCTGGGATGC 3′
		R 5′ GACTTCACTTGTGGCCCAGAT 3′
Beta-actin	ACTB (housekeeping)	F 5′ GAGCGCGGCTACAGCTT 3′
		R 5′ GCCCAATACGACCAAATCCG 3′

F – forward; R – reverse.
